# 2-Hy­droxy-2-(3-oxobutan-2-yl)indan-1,3-dione

**DOI:** 10.1107/S1600536811020253

**Published:** 2011-06-04

**Authors:** Raza Murad Ghalib, Rokiah Hashim, Sayed Hasan Mehdi, Chin Sing Yeap, Hoong-Kun Fun

**Affiliations:** a School of Industrial Technology, Universiti Sains Malaysia, 11800 USM, Penang, Malaysia; b X-ray Crystallography Unit, School of Physics, Universiti Sains Malaysia, 11800 USM, Penang, Malaysia

## Abstract

In the indane ring system of the title mol­ecule, C_13_H_12_O_4_, the hy­droxy-bearing C atom is 0.134 (1) Å out of the plane of the remaining essentially planar atoms (r.m.s. deviation = 0.010 Å). In the crystal, mol­ecules are linked into chains along the *b* axis by inter­molecular O—H⋯O hydrogen bonds. Additional stabilization is provided by weak inter­molecular C—H⋯O hydrogen bonds.

## Related literature

For a related structure, see: Fun *et al.* (2009) ▶. For the stability of the temperature controller used in the data collection, see: Cosier & Glazer (1986 ▶). For ring conformations, see: Cremer & Pople (1975 ▶).
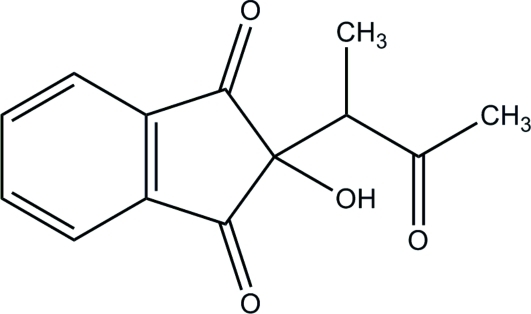


## Experimental

### 

#### Crystal data

C_13_H_12_O_4_
*M* *_r_* = 232.23
Monoclinic, 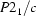

*a* = 9.5080 (5) Å
*b* = 6.7200 (4) Å
*c* = 17.5195 (9) Å
β = 101.044 (1)°
*V* = 1098.66 (10) Å^3^
*Z* = 4
Mo *K*α radiation
μ = 0.10 mm^−1^
*T* = 100 K
0.85 × 0.41 × 0.12 mm

#### Data collection

Bruker APEXII DUO CCD area-detector diffractometer
Absorption correction: multi-scan (*SADABS*; Bruker, 2009 ▶) *T*_min_ = 0.917, *T*_max_ = 0.988
13885 measured reflections
3796 independent reflections
3243 reflections with *I* > 2σ(*I*)
*R*_int_ = 0.025

#### Refinement

*R*[*F* ^2^ > 2σ(*F* ^2^)] = 0.048
*wR*(*F* ^2^) = 0.140
*S* = 1.04
3796 reflections
160 parameters
H atoms treated by a mixture of independent and constrained refinement
Δρ_max_ = 0.58 e Å^−3^
Δρ_min_ = −0.24 e Å^−3^

### 

Data collection: *APEX2* (Bruker, 2009 ▶); cell refinement: *SAINT* (Bruker, 2009 ▶); data reduction: *SAINT*; program(s) used to solve structure: *SHELXTL* (Sheldrick, 2008 ▶); program(s) used to refine structure: *SHELXTL*; molecular graphics: *SHELXTL*; software used to prepare material for publication: *SHELXTL* and *PLATON* (Spek, 2009 ▶).

## Supplementary Material

Crystal structure: contains datablock(s) global, I. DOI: 10.1107/S1600536811020253/lh5255sup1.cif

Structure factors: contains datablock(s) I. DOI: 10.1107/S1600536811020253/lh5255Isup2.hkl

Supplementary material file. DOI: 10.1107/S1600536811020253/lh5255Isup3.cml

Additional supplementary materials:  crystallographic information; 3D view; checkCIF report

## Figures and Tables

**Table 1:** Hydrogen-bond geometry (Å, °)

*D*—H⋯*A*	*D*—H	H⋯*A*	*D*⋯*A*	*D*—H⋯*A*
O2—H1*O*2⋯O1^i^	0.89 (2)	1.90 (2)	2.7880 (13)	171.5 (19)
C12—H12*A*⋯O3^ii^	0.96	2.59	3.5416 (17)	173
C12—H12*B*⋯O3^iii^	0.96	2.52	3.3215 (16)	142

Symmetry codes: (i) 
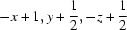
; (ii) 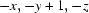; (iii) 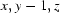.

**Fig. 1.**
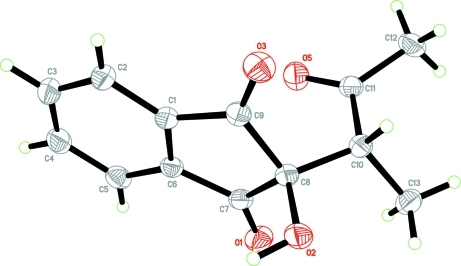
The molecular structure of the title compound with 50% probability ellipsoids for non-H atoms.

**Fig. 2.**
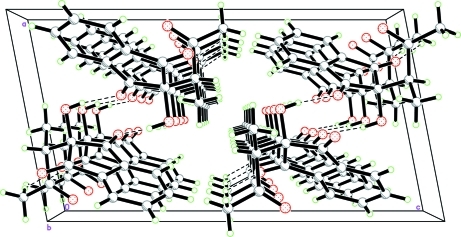
The crystal packing of title compound, viewed down b axis, showing molecules are linked into one-dimensional chains along the b axis. Hydrogen bonds are shown as dashed lines.

## supplementary crystallographic information

### Comment

In the continuation of our studies of ninhydrin reactions with acetone in acidic medium (Fun *et al.*, 2009), we report herein the structure of the title compound (Fig. 1) formed by the reaction of ninhydrin with ethylmethyl ketone in acetic acid. The title compound has potential as a starting material for the synthesis of a large number of complex heterocylic compounds.

In the title compound, the five-membered ring adopts an envelope conformation, with atom C8 forming the flap (Cremer & Pople, 1975). In the indane ring system atom C8 is 0.134 (1)Å out of the plane of the remaining essentially planar atoms. In the crystal structure, the molecules are linked into one-dimensional chains along the *b* axis (Fig. 2) by intermolecular O2—H1O2···O1^i^ and weak C12—H12A···O3^iii^ and C12—H12B···O36^iii^ hydrogen bonds (Table 1).

### Experimental

A mixture of ninhydrin (1.78 g) and ethylmethyl ketome (0.90 ml) in molar ratio 1:1 were heated on water bath for 15 minutes in presence of acetic acid. The reaction mixture was dried on rotatory evaporator at low pressure and crystallized with chloroform-n-hexane (1:1) to give the colorless crystals of the title compound (Yield: 100%, mp. 406–408 K).

### Refinement

The O-bound hydrogen atom was located in a  difference Fourier map and refined freely. The rest of the hydrogen atoms were positioned geometrically [C–H = 0.93–0.98 Å] and refined using a riding model, with *U*_iso_(H) = 1.2 or 1.5 *U*_eq_(C). A rotating-group model was applied for methyl groups.

### Crystal data

**Table:**

C_13_H_12_O_4_	*F*(000) = 488
*M**_r_* = 232.23	*D*_x_ = 1.404 Mg m^−^^3^
Monoclinic, *P*2_1_/*c*	Mo *K*α radiation, λ = 0.71073 Å
Hall symbol: -P 2ybc	Cell parameters from 6185 reflections
*a* = 9.5080 (5) Å	θ = 2.9–32.0°
*b* = 6.7200 (4) Å	µ = 0.10 mm^−^^1^
*c* = 17.5195 (9) Å	*T* = 100 K
β = 101.044 (1)°	Plate, yellow
*V* = 1098.66 (10) Å^3^	0.85 × 0.41 × 0.12 mm
*Z* = 4	

### Data collection

**Table:**

Bruker APEXII DUO CCD area-detector diffractometer	3796 independent reflections
Radiation source: fine-focus sealed tube	3243 reflections with *I* > 2σ(*I*)
graphite	*R*_int_ = 0.025
φ and ω scans	θ_max_ = 32.1°, θ_min_ = 2.2°
Absorption correction: multi-scan (*SADABS*; Bruker, 2009)	*h* = −14→14
*T*_min_ = 0.917, *T*_max_ = 0.988	*k* = −9→9
13885 measured reflections	*l* = −26→26

### Refinement

**Table:**

Refinement on *F*^2^	Primary atom site location: structure-invariant direct methods
Least-squares matrix: full	Secondary atom site location: difference Fourier map
*R*[*F*^2^ > 2σ(*F*^2^)] = 0.048	Hydrogen site location: inferred from neighbouring sites
*wR*(*F*^2^) = 0.140	H atoms treated by a mixture of independent and constrained refinement
*S* = 1.04	*w* = 1/[σ^2^(*F*_o_^2^) + (0.0733*P*)^2^ + 0.4363*P*] where *P* = (*F*_o_^2^ + 2*F*_c_^2^)/3
3796 reflections	(Δ/σ)_max_ < 0.001
160 parameters	Δρ_max_ = 0.58 e Å^−^^3^
0 restraints	Δρ_min_ = −0.24 e Å^−^^3^

### Special details

**Table:**

Experimental. The crystal was placed in the cold stream of an Oxford Cryosystems Cobra open-flow nitrogen cryostat (Cosier & Glazer, 1986) operating at 100.0 (1) K.
Geometry. All e.s.d.'s (except the e.s.d. in the dihedral angle between two l.s. planes) are estimated using the full covariance matrix. The cell e.s.d.'s are taken into account individually in the estimation of e.s.d.'s in distances, angles and torsion angles; correlations between e.s.d.'s in cell parameters are only used when they are defined by crystal symmetry. An approximate (isotropic) treatment of cell e.s.d.'s is used for estimating e.s.d.'s involving l.s. planes.
Refinement. Refinement of *F*^2^ against ALL reflections. The weighted *R*-factor *wR* and goodness of fit *S* are based on *F*^2^, conventional *R*-factors *R* are based on *F*, with *F* set to zero for negative *F*^2^. The threshold expression of *F*^2^ > σ(*F*^2^) is used only for calculating *R*-factors(gt) *etc*. and is not relevant to the choice of reflections for refinement. *R*-factors based on *F*^2^ are statistically about twice as large as those based on *F*, and *R*- factors based on ALL data will be even larger.

### Fractional atomic coordinates and isotropic or equivalent isotropic displacement parameters (Å^2^)

**Table:**

	*x*	*y*	*z*	*U*_iso_*/*U*_eq_	
O1	0.41250 (10)	0.29580 (15)	0.22992 (5)	0.0310 (2)	
O2	0.52668 (8)	0.59788 (15)	0.12770 (5)	0.0277 (2)	
O3	0.25204 (10)	0.81871 (14)	0.05496 (5)	0.0289 (2)	
O5	0.11903 (9)	0.36167 (16)	0.09212 (5)	0.0307 (2)	
C1	0.23418 (11)	0.75422 (17)	0.18829 (6)	0.0200 (2)	
C2	0.15222 (12)	0.90944 (19)	0.20909 (7)	0.0259 (2)	
H2A	0.1228	1.0147	0.1753	0.031*	
C3	0.11597 (13)	0.9011 (2)	0.28246 (7)	0.0303 (3)	
H3A	0.0605	1.0021	0.2978	0.036*	
C4	0.16121 (12)	0.7441 (2)	0.33338 (7)	0.0300 (3)	
H4A	0.1365	0.7431	0.3822	0.036*	
C5	0.24275 (12)	0.5891 (2)	0.31218 (6)	0.0257 (2)	
H5A	0.2725	0.4839	0.3459	0.031*	
C6	0.27822 (10)	0.59662 (17)	0.23882 (6)	0.0202 (2)	
C7	0.36157 (11)	0.45130 (17)	0.20197 (6)	0.0213 (2)	
C8	0.38181 (10)	0.53665 (17)	0.12305 (6)	0.01995 (19)	
C9	0.28310 (11)	0.72095 (17)	0.11399 (6)	0.0202 (2)	
C10	0.34388 (11)	0.40003 (17)	0.05236 (6)	0.0207 (2)	
H10A	0.3536	0.4786	0.0065	0.025*	
C11	0.18853 (11)	0.33382 (17)	0.04139 (6)	0.0219 (2)	
C12	0.12393 (14)	0.2357 (2)	−0.03389 (7)	0.0300 (3)	
H12A	0.0215	0.2329	−0.0393	0.045*	
H12B	0.1596	0.1022	−0.0344	0.045*	
H12C	0.1493	0.3092	−0.0763	0.045*	
C13	0.44326 (12)	0.22016 (19)	0.05498 (7)	0.0273 (2)	
H13A	0.4215	0.1502	0.0064	0.041*	
H13B	0.4296	0.1329	0.0964	0.041*	
H13C	0.5410	0.2645	0.0639	0.041*	
H1O2	0.554 (2)	0.666 (3)	0.1717 (12)	0.044 (5)*	

### Atomic displacement parameters (Å^2^)

**Table:**

	*U*^11^	*U*^22^	*U*^33^	*U*^12^	*U*^13^	*U*^23^
O1	0.0329 (4)	0.0318 (5)	0.0278 (4)	0.0113 (4)	0.0045 (3)	0.0083 (4)
O2	0.0169 (3)	0.0390 (5)	0.0272 (4)	−0.0056 (3)	0.0039 (3)	−0.0064 (4)
O3	0.0332 (4)	0.0278 (4)	0.0256 (4)	0.0009 (4)	0.0059 (3)	0.0069 (3)
O5	0.0240 (4)	0.0371 (5)	0.0319 (4)	−0.0074 (4)	0.0076 (3)	−0.0081 (4)
C1	0.0179 (4)	0.0210 (5)	0.0206 (4)	−0.0004 (3)	0.0026 (3)	−0.0012 (4)
C2	0.0239 (5)	0.0240 (5)	0.0286 (5)	0.0030 (4)	0.0021 (4)	−0.0044 (4)
C3	0.0238 (5)	0.0358 (7)	0.0311 (5)	0.0033 (5)	0.0047 (4)	−0.0116 (5)
C4	0.0228 (5)	0.0438 (7)	0.0239 (5)	−0.0012 (5)	0.0061 (4)	−0.0068 (5)
C5	0.0220 (4)	0.0351 (6)	0.0199 (4)	−0.0018 (4)	0.0035 (3)	0.0008 (4)
C6	0.0168 (4)	0.0238 (5)	0.0194 (4)	−0.0005 (4)	0.0019 (3)	0.0000 (4)
C7	0.0181 (4)	0.0238 (5)	0.0210 (4)	0.0016 (4)	0.0013 (3)	0.0014 (4)
C8	0.0163 (4)	0.0227 (5)	0.0207 (4)	0.0000 (4)	0.0031 (3)	−0.0008 (4)
C9	0.0191 (4)	0.0199 (5)	0.0213 (4)	−0.0023 (3)	0.0031 (3)	0.0001 (4)
C10	0.0196 (4)	0.0220 (5)	0.0206 (4)	0.0000 (4)	0.0044 (3)	−0.0001 (4)
C11	0.0203 (4)	0.0200 (5)	0.0239 (4)	0.0011 (4)	0.0003 (3)	−0.0009 (4)
C12	0.0292 (5)	0.0312 (6)	0.0256 (5)	0.0000 (5)	−0.0046 (4)	−0.0044 (5)
C13	0.0232 (5)	0.0265 (6)	0.0323 (5)	0.0031 (4)	0.0057 (4)	−0.0021 (4)

### Geometric parameters (Å, °)

**Table:**

O1—C7	1.2147 (14)	C5—H5A	0.9300
O2—C8	1.4249 (12)	C6—C7	1.4815 (15)
O2—H1O2	0.89 (2)	C7—C8	1.5425 (15)
O3—C9	1.2129 (13)	C8—C10	1.5283 (15)
O5—C11	1.2193 (14)	C8—C9	1.5436 (15)
C1—C2	1.3917 (16)	C10—C11	1.5192 (15)
C1—C6	1.3924 (15)	C10—C13	1.5294 (16)
C1—C9	1.4808 (14)	C10—H10A	0.9800
C2—C3	1.3941 (17)	C11—C12	1.4969 (15)
C2—H2A	0.9300	C12—H12A	0.9600
C3—C4	1.395 (2)	C12—H12B	0.9600
C3—H3A	0.9300	C12—H12C	0.9600
C4—C5	1.3908 (18)	C13—H13A	0.9600
C4—H4A	0.9300	C13—H13B	0.9600
C5—C6	1.3906 (14)	C13—H13C	0.9600
			
C8—O2—H1O2	108.3 (13)	C10—C8—C9	110.74 (8)
C2—C1—C6	121.25 (10)	C7—C8—C9	102.29 (8)
C2—C1—C9	129.00 (10)	O3—C9—C1	126.98 (10)
C6—C1—C9	109.71 (9)	O3—C9—C8	124.49 (10)
C1—C2—C3	117.45 (11)	C1—C9—C8	108.53 (9)
C1—C2—H2A	121.3	C11—C10—C8	110.56 (8)
C3—C2—H2A	121.3	C11—C10—C13	110.64 (9)
C2—C3—C4	121.30 (11)	C8—C10—C13	113.70 (9)
C2—C3—H3A	119.3	C11—C10—H10A	107.2
C4—C3—H3A	119.3	C8—C10—H10A	107.2
C5—C4—C3	120.98 (11)	C13—C10—H10A	107.2
C5—C4—H4A	119.5	O5—C11—C12	121.44 (10)
C3—C4—H4A	119.5	O5—C11—C10	120.83 (10)
C6—C5—C4	117.77 (11)	C12—C11—C10	117.73 (10)
C6—C5—H5A	121.1	C11—C12—H12A	109.5
C4—C5—H5A	121.1	C11—C12—H12B	109.5
C5—C6—C1	121.24 (10)	H12A—C12—H12B	109.5
C5—C6—C7	128.57 (10)	C11—C12—H12C	109.5
C1—C6—C7	110.18 (9)	H12A—C12—H12C	109.5
O1—C7—C6	126.63 (10)	H12B—C12—H12C	109.5
O1—C7—C8	124.94 (10)	C10—C13—H13A	109.5
C6—C7—C8	108.34 (9)	C10—C13—H13B	109.5
O2—C8—C10	107.17 (8)	H13A—C13—H13B	109.5
O2—C8—C7	109.96 (8)	C10—C13—H13C	109.5
C10—C8—C7	116.90 (9)	H13A—C13—H13C	109.5
O2—C8—C9	109.65 (9)	H13B—C13—H13C	109.5
			
C6—C1—C2—C3	0.02 (17)	C2—C1—C9—O3	−5.20 (19)
C9—C1—C2—C3	177.51 (11)	C6—C1—C9—O3	172.52 (11)
C1—C2—C3—C4	0.58 (18)	C2—C1—C9—C8	175.17 (11)
C2—C3—C4—C5	−0.79 (19)	C6—C1—C9—C8	−7.10 (12)
C3—C4—C5—C6	0.37 (18)	O2—C8—C9—O3	73.28 (13)
C4—C5—C6—C1	0.22 (16)	C10—C8—C9—O3	−44.77 (14)
C4—C5—C6—C7	−179.24 (11)	C7—C8—C9—O3	−170.06 (10)
C2—C1—C6—C5	−0.43 (16)	O2—C8—C9—C1	−107.08 (9)
C9—C1—C6—C5	−178.36 (10)	C10—C8—C9—C1	134.86 (9)
C2—C1—C6—C7	179.12 (10)	C7—C8—C9—C1	9.58 (11)
C9—C1—C6—C7	1.19 (12)	O2—C8—C10—C11	−177.89 (9)
C5—C6—C7—O1	1.15 (19)	C7—C8—C10—C11	58.23 (12)
C1—C6—C7—O1	−178.35 (11)	C9—C8—C10—C11	−58.34 (11)
C5—C6—C7—C8	−175.32 (10)	O2—C8—C10—C13	56.96 (12)
C1—C6—C7—C8	5.18 (12)	C7—C8—C10—C13	−66.92 (12)
O1—C7—C8—O2	−68.98 (14)	C9—C8—C10—C13	176.52 (9)
C6—C7—C8—O2	107.57 (10)	C8—C10—C11—O5	−12.09 (15)
O1—C7—C8—C10	53.47 (14)	C13—C10—C11—O5	114.77 (12)
C6—C7—C8—C10	−129.99 (9)	C8—C10—C11—C12	167.27 (10)
O1—C7—C8—C9	174.59 (11)	C13—C10—C11—C12	−65.87 (13)
C6—C7—C8—C9	−8.87 (10)		

### Hydrogen-bond geometry (Å, °)

**Table:**

*D*—H···*A*	*D*—H	H···*A*	*D*···*A*	*D*—H···*A*
O2—H1O2···O1^i^	0.89 (2)	1.90 (2)	2.7880 (13)	171.5 (19)
C12—H12A···O3^ii^	0.96	2.59	3.5416 (17)	173
C12—H12B···O3^iii^	0.96	2.52	3.3215 (16)	142

Symmetry codes: (i) −*x*+1, *y*+1/2, −*z*+1/2; (ii) −*x*, −*y*+1, −*z*; (iii) *x*, *y*−1, *z*.

## References

[bb1] Bruker (2009). *APEX2*, *SAINT* and *SADABS* Bruker AXS Inc., Madison, Wisconsin, USA.

[bb2] Cosier, J. & Glazer, A. M. (1986). *J. Appl. Cryst.* **19**, 105–107.

[bb3] Cremer, D. & Pople, J. A. (1975). *J. Am. Chem. Soc.* **97**, 1354–1358.

[bb4] Fun, H.-K., Quah, C. K., Parveen, M., Ghalib, R. M. & Mehdi, S. H. (2009). *Acta Cryst.* E**65**, o1209. 10.1107/S1600536809016067 PMC2969680 21583078

[bb5] Sheldrick, G. M. (2008). *Acta Cryst.* A**64**, 112–122. 10.1107/S0108767307043930 18156677

[bb6] Spek, A. L. (2009). *Acta Cryst.* D**65**, 148–155. 10.1107/S090744490804362X PMC2631630 19171970

